# Electrochemical and Optical Biosensors for Acetylcholine Monitoring in Neurodegeneration

**DOI:** 10.3390/mi17101122

**Published:** 2026-09-25

**Authors:** Juan Pablo Hervás-Pérez, Laura Muñoz-Oporto, María Moreno-Guzmán, Marta Sánchez-Paniagua

**Affiliations:** Department of Chemistry in Pharmaceutical Sciences, Faculty of Pharmacy, Complutense University of Madrid, 28040 Madrid, Spain; jphervas@ucm.es (J.P.H.-P.); lamuno08@ucm.es (L.M.-O.); marimore@ucm.es (M.M.-G.)

**Keywords:** acetylcholine, electrochemical biosensing, optical biosensing, neurodegenerative diseases, Alzheimer’s disease, neurotransmitter monitoring, nanomaterials, point-of-care diagnostics, cholinergic dysfunction

## Abstract

Acetylcholine (ACh) plays a pivotal role in cognition, learning, memory, and neuronal plasticity, and alterations in cholinergic neurotransmission are closely associated with neurodegenerative disorders, particularly Alzheimer’s disease and related dementias. Consequently, the development of analytical tools capable of accurately monitoring ACh has attracted considerable interest in both neuroscience and clinical research. However, the determination of ACh remains challenging because of its low physiological concentrations, rapid enzymatic degradation, and the complexity of biological matrices. This review provides a comprehensive overview of recent advances in electrochemical and optical biosensors developed for ACh detection, with particular emphasis on progress reported between 2020 and 2026. The sensing principles, analytical performance, and technological characteristics of enzymatic and non-enzymatic electrochemical platforms, as well as colorimetric, fluorescent, surface plasmon resonance, and emerging molecular optical sensors, are critically discussed. Particular attention is given to the role of nanomaterials, signal-amplification strategies, miniaturized devices, wearable and implantable platforms, and approaches enabling real-time monitoring. Recent studies demonstrate remarkable improvements in sensitivity and detection limits; however, translation towards clinical applications remains limited by challenges related to selectivity in complex biological samples, biofouling, long-term stability, reproducibility, and the scarcity of validation studies involving authentic patient-derived specimens. Overall, current advances indicate a shift from proof-of-concept sensor development towards application-oriented platforms aimed at continuous neurochemical monitoring and point-of-care analysis. Future progress will depend on achieving robust performance in physiologically relevant environments and establishing clinically validated biosensing strategies for the assessment of cholinergic dysfunction in neurodegenerative diseases.

## 1. Background and Motivation

Neurotransmitters are chemical messengers responsible for transmitting signals from presynaptic to postsynaptic cells, thereby regulating essential physiological functions such as cognition, movement, learning, and memory [1,2,3]. Acetylcholine (ACh) is one of the most important neurotransmitters in both vertebrates and invertebrates due to its widespread distribution and diverse physiological functions. ACh is synthesized by the enzyme choline acetyltransferase (ChAT) through the transfer of an acetyl group from acetyl coenzyme A to a choline molecule. In mammals, ACh is present in both the central nervous system (CNS) and the peripheral nervous system (PNS), exerting its effects through the activation of nicotinic and muscarinic ACh receptors [4,5,6]. Within the CNS, ACh plays a fundamental role in neuronal plasticity and in the modulation of multiple neural networks involved in attention, learning, motivation, emotional processing, and all stages of memory processing, including memory formation, consolidation, and retrieval [7,8]. Alterations in cholinergic neurotransmission have been associated with cognitive impairment and with the progression of several neurodegenerative disorders, particularly Alzheimer’s disease and other forms of dementia [8,9,10,11].

The strong relationship between cholinergic dysfunction and cognitive decline has highlighted the importance of ACh quantification as a potential biomarker for evaluating disease progression and monitoring therapeutic responses [9,10,12,13]. However, the analytical determination of ACh remains particularly challenging. These difficulties arise from several intrinsic characteristics of the neurotransmitter, including its extremely low physiological concentrations, typically in the nanomolar range, its rapid enzymatic degradation by acetylcholinesterase (AChE), and the complexity of biological matrices such as blood, plasma, serum, cerebrospinal fluid, urine, and tissues [8,14].

A wide variety of analytical methods have been developed for their determination, and these methodologies have evolved considerably over recent decades. Classical approaches include enzymatic assays based on coupled reactions that enable indirect detection of the neurotransmitter [14]. Chromatographic techniques, particularly high-performance liquid chromatography (HPLC) coupled with electrochemical detection [14,15,16] or mass spectrometry [8,17,18], have become the reference methods for ACh determination, providing high sensitivity, selectivity, and the capability for simultaneous analysis of multiple analytes [8,14]. Advanced spectroscopic techniques such as surface-enhanced Raman spectroscopy (SERS) have also attracted attention because of their ability to amplify Raman signals using nanostructured substrates, thereby enabling the detection of analytes at extremely low concentrations [8,19,20,21].

Other approaches, including microdialysis, have enabled investigations of ACh release dynamics in vivo in animal models [8]. In parallel, biosensors, particularly those based on enzymes such as AChE and choline oxidase (ChOx), have emerged as versatile analytical tools for ACh monitoring, driven by advances in nanostructured materials and miniaturized sensing platforms [22,23,24]. More recently, genetically encoded fluorescent sensors have opened new opportunities for real-time monitoring of cholinergic activity in living organisms [7].

Among the different analytical approaches currently available, biosensors have attracted particular attention because they combine high sensitivity, rapid response times, portability, and compatibility with point-of-care and real-time monitoring applications. Electrochemical biosensors offer several advantages, including low cost, miniaturization capability, and excellent analytical sensitivity, making them suitable for vivo and clinical applications. Optical biosensors, including fluorescence- and plasmon-based platforms, provide complementary advantages such as high selectivity, multiplexing capability, and non-invasive detection strategies. The integration of nanomaterials, microfabrication technologies, and advanced signal amplification methods has further improved the analytical performance of these devices and expanded their potential applications in neurochemical monitoring [25,26,27].

In this context, this review provides a comprehensive overview of electrochemical and optical biosensors developed for the detection and quantification of ACh. Their sensing principles, analytical performance, advantages, limitations, and emerging applications are discussed. A particular emphasis is placed on their potential role in investigating cholinergic dysfunction and in supporting the diagnosis and monitoring of cognitive impairment associated with neurodegenerative diseases.

To identify relevant studies, a comprehensive literature search was conducted using the Web of Science, Scopus, PubMed, and ScienceDirect databases. The search strategy included combinations of the keyword’s “acetylcholine”, “biosensor”, “electrochemical biosensor”, “optical biosensor”, “wearable biosensor”, “implantable biosensor”, “fluorescent sensor”, “colorimetric sensor”, and “surface plasmon resonance”. Particular emphasis was placed on studies published between 2020 and 2026, while seminal earlier publications were also considered when they provided important foundations for the development of ACh biosensing technologies. Studies were selected according to their relevance to ACh detection principles, analytical performance, technological development, and biomedical applications.

## 2. Acetylcholine and Neurodegeneration: Clinical and Pathophysiological Significance

ACh is one of the most important neurotransmitters in both the central and peripheral nervous systems due to its broad distribution and the diversity of physiological processes it regulates. In the central nervous system, cholinergic neurotransmission plays a crucial role in synaptic plasticity and in the modulation of multiple neuronal networks involved in cognition and behavior. Cholinergic signaling contributes to the regulation of attention, learning, motivation, emotional processing, appetite, sleep–wake cycles, and stress responses, while also participating in all stages of memory processing, including memory encoding, consolidation, and retrieval [4,7,8].

Among the different cholinergic pathways in the brain, the basal forebrain cholinergic system is considered particularly important for cognitive function. Cholinergic neurons located in the medial septum, diagonal band of Broca, and nucleus basalis of Meynert project extensively to the hippocampus, amygdala, and cerebral cortex, where they modulate neuronal excitability and synaptic transmission. Alterations in these pathways have been consistently associated with deficits in memory, executive function, and attention [10,11]. Normal aging is accompanied by progressive changes in cholinergic neurotransmission, including reductions in cholinergic neuron function, decreased ChAT activity, and alterations in ACh receptor expression. These changes are thought to contribute to age-associated cognitive decline and increased vulnerability to neurodegenerative disorders [11]. However, cholinergic dysfunction is considerably more pronounced in pathological conditions and has become one of the hallmarks of several neurodegenerative diseases.

The strongest evidence supporting the involvement of cholinergic dysfunction in neurodegeneration comes from Alzheimer’s disease (AD). One of the earliest and most characteristic pathological events in AD is the degeneration of cholinergic neurons located in the nucleus basalis of Meynert (Ch4 cell group) within the basal forebrain, resulting in a substantial reduction in cortical cholinergic innervation. This neuronal loss is accompanied by decreased ChAT activity and alterations in both nicotinic and muscarinic ACh receptors, ultimately leading to impaired cholinergic neurotransmission [11,14].

Importantly, the severity of cholinergic degeneration correlates with the degree of cognitive impairment and dementia progression observed in AD patients [9]. Reported ACh concentrations vary substantially depending on the biological compartment and analytical approach employed. In human serum, a recent study reported ACh concentrations ranging from 9.0 to 12.0 nM in apparently healthy individuals, whereas concentrations of 1.0–6.5 nM were measured in patients with Alzheimer’s disease using an enzymatic biosensor [22]. These values should be interpreted as study-specific measurements rather than universally established physiological or pathological reference ranges, given the limited sample size and the potential influence of sampling, storage, and analytical conditions. At the neuronal level, ACh concentrations are considerably higher during synaptic transmission. For example, nanoscale electroanalytical measurements in Aplysia californica neurons have reported local concentrations of up to 2.4 mM in the immediate vicinity of the synapse [28]. These findings highlight the strong dependence of ACh concentration on the biological compartment and emphasize the need for biosensors with an appropriate dynamic range tailored to their intended application. Whereas healthy individuals typically exhibit average ACh concentrations of approximately 9 nM, concentrations below 6 nM have been reported in patients with Alzheimer’s disease [14]. This reduction in neurotransmitter availability, together with the critical role of cholinergic signaling in memory formation, gave rise to the cholinergic hypothesis of Alzheimer’s disease, which remains one of the most robust and extensively investigated hypotheses explaining disease pathogenesis [29].

The cholinergic hypothesis proposes that impaired cholinergic neurotransmission contributes substantially to the memory deficits and cognitive decline characteristic of Alzheimer’s disease (AD) [9]. Initial support for this hypothesis came from pharmacological studies demonstrating that administration of scopolamine, a centrally acting muscarinic ACh receptor antagonist, induces transient impairments in memory and attention in healthy individuals, providing a pharmacological model of cholinergic dysfunction relevant to the cognitive deficits observed in AD [30,31,32], Subsequent post-mortem studies demonstrated marked reductions in cholinergic markers, particularly ChAT activity, in the brains of patients with AD, providing further evidence for the involvement of cholinergic dysfunction in cognitive impairment [9,33,34,35]. Together, pharmacological, neurochemical, and neuropathological findings have provided substantial support for the contribution of cholinergic dysfunction to the cognitive manifestations of AD, although this dysfunction is now considered one component of a broader and multifactorial disease process.

More recently, neuroimaging approaches, particularly positron emission tomography (PET), have provided in vivo evidence of alterations in the cholinergic system in AD. PET studies using radioligands targeting cholinergic receptors have demonstrated reduced availability of α4β2 nicotinic ACh receptors in brain regions critically involved in cognition, including the basal forebrain, hippocampus, and frontotemporal cortices, with the magnitude of these alterations being associated with cognitive impairment [9,36]. However, these alterations are not necessarily uniform across brain regions, receptor subtypes, or stages of disease, and differences between PET studies may partly reflect methodological differences in radioligand properties and quantitative approaches.

Beyond Alzheimer’s disease, cholinergic dysfunction has also been implicated in several other neurodegenerative disorders. In Parkinson’s disease, degeneration of cholinergic projections contributes to cognitive impairment and increases the risk of developing Parkinson’s disease dementia [37,38]. Similarly, patients with dementia with Lewy bodies exhibit severe cholinergic deficits that are believed to contribute to attentional dysfunction, visual hallucinations, and cognitive decline [39,40]. Cholinergic alterations have also been described in frontotemporal dementia and vascular dementia, although their contribution to disease progression appears to be more heterogeneous than in Alzheimer’s disease [8,37].

The close relationship between cholinergic dysfunction and cognitive decline highlights the potential value of ACh and related cholinergic markers as biomarkers of neurodegenerative disease. Measurements of ACh concentrations in biological fluids, particularly cerebrospinal fluid (CSF), may provide information about the status of central cholinergic neurotransmission and its relationship with cognitive impairment. Moreover, ACh levels could provide valuable information regarding disease progression, therapeutic response, and treatment efficacy, particularly in patients receiving cholinesterase inhibitors or other cholinergic therapies [12,13]. Consequently, the development of analytical techniques capable of rapidly and sensitively quantifying ACh in biological matrices represents an important objective in both clinical diagnostics and neuroscience research [18].

## 3. Analytical Approaches for Acetylcholine Detection

The analytical determination of ACh has attracted considerable interest over recent decades due to the central role of cholinergic neurotransmission in cognition, memory, and neurodegenerative diseases. Reliable determination of ACh has become a major focus in analytical chemistry because of the need to accurately monitor this neurotransmitter in complex biological systems. The analysis of ACh is particularly demanding due to its low endogenous abundance, rapid hydrolysis by AChE, and the presence of numerous potentially interfering compounds in biological matrices, including plasma, cerebrospinal fluid, urine, and brain tissue [8,18]. To address these challenges, a wide range of analytical techniques have been proposed, aiming to enhance detection sensitivity, improve selectivity, and enable more reliable and, in some cases, real-time quantification of ACh [14,27].

### 3.1. Enzymatic Assay

Among the earliest approaches used for ACh determination are enzymatic assays based on coupled enzymatic reactions. These methods generally rely on the hydrolysis of ACh by AChE to produce choline and acetate, followed by the oxidation of choline by ChOx, generating hydrogen peroxide as a secondary product that can subsequently be quantified by colorimetric, fluorimetric, or electrochemical methods [14,41].

The simplicity, low cost, and relatively short analysis times associated with enzymatic assays have made them attractive for routine laboratory applications. Nevertheless, these methods are indirect and are often affected by matrix interferences, limited enzyme stability, and analytical sensitivity when compared with more advanced analytical techniques [42]. Furthermore, the requirement for multiple enzymatic steps may introduce variability and reduce analytical reproducibility [14].

### 3.2. Chromatographic Techniques

Chromatographic techniques currently represent the most widely established methods for quantitative determination of ACh in biological samples due to their excellent sensitivity and selectivity.

High-performance liquid chromatography coupled with electrochemical detection (HPLC-ED) has become one of the most extensively employed analytical methods for the determination of ACh in biological matrices [8,14,43,44]. In these systems, chromatographic separation is typically followed by enzymatic conversion of ACh into hydrogen peroxide, which is subsequently detected amperometrically. HPLC-ED offers high analytical sensitivity and has been widely applied for the analysis of brain microdialysates, cerebrospinal fluid, and tissue extracts. In particular, the technique has played a fundamental role in preclinical studies investigating cholinergic neurotransmission and the effects of pharmacological interventions targeting the cholinergic system [8]. However, HPLC-ED requires relatively expensive instrumentation, extensive sample preparation procedures, and trained personnel, limiting its applicability for point-of-care testing or real-time monitoring applications.

The development of liquid chromatography coupled to mass spectrometry (LC-MS and LC-MS/MS) has represented a major advance in neurotransmitter analysis by enabling highly sensitive and selective quantification of multiple analytes simultaneously [8,45]. LC-MS/MS methods allow direct identification of ACh based on its mass-to-charge ratio while minimizing interference from endogenous compounds present in complex biological matrices [18,46]. Compared with HPLC-ED, LC-MS/MS provides superior specificity and multiplexing capabilities, allowing the simultaneous determination of ACh together with other neurotransmitters and metabolites involved in neurodegenerative processes [18,46,47].

Consequently, LC-MS/MS has become an important reference approach for targeted neurochemical profiling studies. Nevertheless, the high cost of instrumentation, complex sample preparation procedures, and lack of portability remain significant limitations for routine clinical implementation [8].

### 3.3. Spectroscopic Approaches

Spectroscopic techniques have also been investigated as alternative approaches for ACh determination because of their potential for rapid and sensitive analysis. Among these techniques, surface-enhanced Raman spectroscopy (SERS) has emerged as a promising strategy for ACh detection owing to the enhancement of Raman scattering provided by nanostructured metallic substrates. In particular, gold- and silver-based nanostructures have been explored to improve the sensitivity of Raman-based detection, enabling the analysis of ACh at low concentrations [8,48,49].

Recent developments in nanostructured SERS platforms have further improved the analytical performance of ACh detection, with recent studies demonstrating high sensitivity in both standard solutions and biological samples [48,49]. These approaches highlight the potential of Raman-based techniques for sensitive ACh analysis, although their application remains less established than chromatographic and electrochemical methods.

Other spectroscopic techniques, including fluorescence spectroscopy and nuclear magnetic resonance (NMR) spectroscopy, have also been investigated for ACh determination. Fluorescence-based approaches have demonstrated high sensitivity and selectivity for ACh detection, including strategies based on enzymatic reactions and fluorescent probes [50,51]. NMR-based approaches have also been explored for the detection and monitoring of ACh in biological systems, including the analysis of ACh synthesis and its presence in neuronal tissue [52,53]. However, their application remains considerably less widespread than chromatographic and electrochemical methods because of limitations related to sensitivity, selectivity, instrumental complexity, or the need for specialized experimental configurations [8].

### 3.4. In Vivo Monitoring Techniques

The determination of neurotransmitters in vivo enables the direct correlation of neurochemical dynamics with neuronal activity and behavior while preserving intact neuronal circuits [7,54]. Consequently, in vivo analytical techniques have become highly attractive for monitoring ACh concentrations under physiological conditions and in neurodegenerative disorders.

Brain microdialysis remains one of the most widely employed methods for in vivo determination of ACh. The technique is based on the continuous collection of extracellular fluid through a semi-permeable membrane implanted into a specific brain region [7,54]. The recovered dialysate is subsequently analyzed using complementary analytical techniques such as HPLC-ED or LC-MS/MS. Microdialysis probes require careful optimization of membrane length, membrane diameter, and perfusion flow rates, as these parameters strongly influence ACh recovery efficiency. In addition, the perfusion solution commonly contains AChE inhibitors to minimize enzymatic degradation of the neurotransmitter and improve recovery rates [7], Despite its widespread use, microdialysis suffers from relatively poor temporal resolution because the measured signal represents an average neurotransmitter concentration over the sampling period rather than rapid fluctuations in synaptic release. Furthermore, the invasive nature of probe implantation may induce local tissue damage and alter the neurochemical environment surrounding the sampling region [7,54].

Positron emission tomography (PET) has become an important tool for investigating the cholinergic system in vivo through radiolabeled tracers targeting molecular components involved in cholinergic neurotransmission, including muscarinic and nicotinic receptors, the vesicular ACh transporter (VAChT), and AChE [55]. PET studies have revealed alterations in cholinergic function in neurodegenerative disorders such as Alzheimer’s disease, with reductions in AChE activity reported particularly in the amygdala and cerebral cortex [56]. However, PET provides an indirect assessment of cholinergic neurotransmission because the radiotracers target receptors, transporters, or enzymes rather than measuring the concentration and rapid dynamics of ACh itself. Characterizing synaptic ACh release at high temporal and spatial resolution therefore requires complementary techniques capable of directly monitoring neurotransmitter dynamics, including electrochemical and other biosensing approaches [28].

Recent developments in D-shaped photonic crystal fiber SPR architectures have demonstrated that simplified geometries combined with dual plasmonic layers (Au/TiO_2_) can achieve exceptionally high wavelength sensitivities—up to ~34,400 nm/RIU for acetylcholine detection—while remaining compatible with practical fabrication routes. These platforms also integrate ANN-based refractive index prediction, further improving analytical precision in biochemical sensing [57]. Overall, conventional analytical methods provide excellent sensitivity and selectivity for ACh determination and have played a fundamental role in the characterization of cholinergic neurotransmission. However, their limited portability, high instrumentation costs, extensive sample preparation, specialized instrumentation requirements, and limited suitability for continuous and real-time monitoring have stimulated the development of biosensor technologies [8,27]. By combining biological recognition elements with miniaturized transducers, biosensors offer rapid, sensitive, and potentially point-of-care detection of ACh, making them particularly attractive for neurochemical monitoring and applications related to neurological and neurodegenerative disorders [26,27]. The following section therefore focuses on electrochemical and optical biosensor platforms for ACh detection, with particular emphasis on the technological strategies developed to improve sensitivity, selectivity, stability, miniaturization, and real-time monitoring capabilities.

## 4. Electrochemical and Optical Biosensors for ACh Detection

The rapid evolution of ACh biosensors during the last decade has extended beyond improvements in analytical sensitivity, reflecting a broader transition toward sensing platforms capable of addressing increasingly complex biomedical challenges. As illustrated in Figure 1, the development of ACh biosensors can be broadly viewed as a progression from conventional enzyme-based platforms toward increasingly advanced architectures designed to improve analytical performance, stability, miniaturization, and applicability to real-time and in vivo monitoring. More recent developments have further emphasized wearable and implantable configurations, continuous monitoring, and validation in biologically relevant samples, highlighting the growing interest in translating ACh biosensing technologies toward clinically relevant applications. The following sections discuss these developments according to their underlying sensing principles, beginning with the general principles of ACh biosensors and subsequently addressing electrochemical and optical platforms.

### 4.1. General Principles of ACh Biosensors

A biosensor combines a biological recognition element with a physicochemical transducer that converts the recognition event or the biochemical reaction associated with the analyte into a measurable signal [58,59]. In the case of ACh, the biorecognition element is predominantly enzymatic, with AChE being the most widely employed enzyme due to its high catalytic specificity toward ACh. AChE hydrolyzes ACh into choline and acetate, providing the primary biochemical reaction underlying enzymatic ACh biosensing. In many ACh biosensors, ChO is incorporated as an auxiliary enzyme to further oxidize the choline generated by AChE, producing hydrogen peroxide (H_2_O_2_) as a detectable reaction product. This AChE/ChO enzymatic configuration has been widely employed for ACh detection [60].

It is worth distinguishing enzymatic biosensors from other sensor approaches reported for ACh determination. Non-enzymatic sensors, including platforms based on metal or metal-oxide nanomaterials and other catalytic interfaces, have also been developed for direct ACh detection. Although these systems are frequently described as “non-enzymatic ACh biosensors” in the literature, they do not contain a biological recognition element and are therefore considered here as ACh sensors rather than biosensors in the strict sense. This distinction is particularly relevant when comparing the different sensing strategies reported for ACh.

From a transduction perspective, ACh biosensors reported in the literature can mainly be classified into electrochemical and optical platforms [27]. Electrochemical transduction converts the biochemical reaction into an electrical response, including changes in current, potential, or interfacial impedance [27]. Optical ACh biosensors, in contrast, translate the biochemical reaction or recognition event into a measurable optical response, with colorimetric, fluorescence, and surface plasmon resonance (SPR) approaches being among the principal strategies reported for ACh detection [14,26,61].

Additional advances in LSPR-based fiber probes demonstrate that depositing Ti_3_C_2_ MXene nanosheets onto GNR-functionalized fibers increases refractive index sensitivity by ~44%, owing to MXene’s hydrophilicity, broadband absorption and enlarged evanescent field depth. These hybrid LSPR sensors achieve linear acetylcholine detection up to 900 µM with sensitivities around 0.03 nm/µM [62].

Other transduction mechanisms have also been demonstrated, although they have received considerably less attention. In particular, piezoelectric quartz crystal microbalance (QCM) platforms have been coupled with enzymatic ACh recognition. An early example employed a multilayer AChE/ChO/horseradish peroxidase system, in which the enzymatic conversion of ACh led to the formation of an insoluble product whose accumulation on the quartz crystal was monitored through changes in resonance frequency [63]. Thus, piezoelectric transduction represents a demonstrated but comparatively minor approach for ACh sensing [63].

Overall, the recent ACh sensing literature is dominated by electrochemical and optical platforms, owing to their high sensitivity, rapid response, relatively simple instrumentation, and compatibility with miniaturized sensing architectures. These characteristics are particularly relevant for ACh detection, where low endogenous concentrations, rapid enzymatic degradation, and complex biological matrices impose stringent requirements on sensor performance. Recent optical fiber SPR platforms incorporating Ti_3_C_2_ MXene and gold nanorods via electrostatic layer-by-layer assembly have shown synergistic enhancement of evanescent field penetration and local electromagnetic amplification, improving refractive index sensitivity by more than 200%. These MXene–GNR hybrid probes enable selective acetylcholine detection with response times around 220 s and detection limits in the low-micromolar range [64]. Despite their analytical advantages, ACh biosensors still face challenges related to enzyme stability, operational lifetime, matrix effects in complex biological samples, biofouling, and interference from electroactive or optically active species. Consequently, recent research has focused on improving bioreceptor immobilization, engineering nanostructured sensing interfaces, introducing antifouling strategies, and implementing signal-amplification approaches to enhance sensitivity, selectivity, and operational stability [22,26].

Accordingly, the following sections focus on these two major classes of ACh biosensors, electrochemical and optical approaches.

### 4.2. Electrochemical Biosensors

Electrochemical biosensors represent one of the most extensively investigated sensing strategies for ACh detection because of their high analytical sensitivity, rapid response times, relatively simple instrumentation, and compatibility with miniaturized and implantable platforms [25,26]. In recent years, the development of electrochemical ACh biosensors has progressively shifted from proof-of-concept analytical devices toward platforms designed to address more demanding biomedical applications. Early studies primarily focused on enzyme immobilization and the development of modified electrode surfaces to improve analytical sensitivity, whereas subsequent approaches incorporated nanostructured materials to enhance electrode performance. More recent developments have included graphene derivatives, metal nanoparticles, metal oxides, conductive polymers, and nanozyme-based architectures, which can improve electrode surface area, electron transfer, catalytic activity, and signal amplification [26,27]. At the same time, increasing attention has been directed toward operational stability, miniaturization, flexibility, and compatibility with physiologically relevant environments, reflecting the growing interest in electrochemical ACh biosensors for real-time and potentially in vivo neurochemical monitoring [27].

Depending on the sensing configuration, ACh has been determined using amperometric, voltametric, potentiometric, and impedimetric approaches. In enzymatic systems, amperometric and voltametric detection commonly relies on the electrochemical oxidation or reduction in H_2_O_2_ generated by the AChE/ChO cascade, whereas impedance-based approaches monitor changes in interfacial electron-transfer properties.

#### 4.2.1. Enzymatic Electrochemical Biosensors

A large proportion of electrochemical biosensors reported for ACh determination are enzymatic devices based on AChE, frequently coupled with ChO. Following ACh hydrolysis by AChE, the resulting choline is oxidized by ChO, generating betaine aldehyde and hydrogen peroxide (H_2_O_2_). The H_2_O_2_ generated in this secondary reaction provides a readily detectable product that can be coupled to electrochemical transduction. Because the amount of H_2_O_2_ generated is related to the initial ACh concentration, its electrochemical detection enables quantitative determination of ACh, most commonly through amperometric measurements.

The main advantages of enzymatic biosensors include their excellent selectivity and low detection limits. However, their analytical performance is strongly influenced by environmental factors such as temperature, pH, and ionic strength, while enzyme denaturation can significantly reduce long-term stability and reproducibility [14].

Recent developments have focused on improving enzyme immobilization strategies and electron transfer efficiencies through the incorporation of nanostructured materials. Early studies demonstrated that ChOx entrapped within polyacrylamide microgels could provide stable amperometric responses while preserving enzymatic activity, anticipating many of the immobilization strategies employed in current nanostructured ACh biosensors [65]. Other examples include carbon paste electrodes functionalized with dendrimers [66], platinum nanoparticle-decorated graphene oxide composites [22], copper nanozyme-reduced graphene oxide hybrids [67], and conductive polymer nanofibers based on polypyrrole–chitosan composites [68].

An important aspect that emerges from the recent literature is the type of biological matrices used for sensor validation. Despite the remarkable analytical performances reported for many electrochemical platforms, most studies have been limited to standard ACh solutions or artificially enriched biological samples rather than authentic clinical specimens. For example, device performance was assessed in some studies exclusively using standard ACh solutions [69,70], whereas others employed human serum samples supplemented with known concentrations of ACh to provide a more representative biological matrix [67,68,71]. Similarly, validation studies were performed using artificial blood matrices designed to closely mimic physiological conditions [66].

Only a limited number of studies have progressed toward biologically and clinically relevant samples. Successful measurements were achieved in rat blood and brain tissue, and subsequent evaluation following device implantation into rat brain tissue represented an important advance towards in vivo neurochemical monitoring [72]. More recently, electrochemical biosensors have been validated using human biopsy samples obtained during surgery from patients with Hirschsprung disease [73], while validation in serum samples collected from patients with Alzheimer’s disease has further extended their potential clinical applicability [26].

This progression from standard solutions to complex biological matrices illustrates the gradual maturation of ACh biosensing technologies. Nevertheless, the scarcity of studies involving human clinical samples remains one of the major barriers preventing the translation of ACh biosensors into routine diagnostic and monitoring applications for neurodegenerative diseases.

Among the enzymatic electrochemical biosensors reported in recent years, particular attention should be given to a platform that represents one of the earliest attempts to advance ACh biosensing from proof-of-concept demonstrations towards clinically relevant applications [22,26]. The device consisted of a pencil graphite electrode (PGE) modified with platinum nanoparticles (PtNPs) electrodeposited onto a porous graphene oxide nanosheet (GONS) composite. AChE and ChOx nanoparticles were subsequently co-immobilized onto the nanostructured surface, enabling the enzymatic cascade in which ACh is hydrolyzed to choline, followed by oxidation of choline to generate hydrogen peroxide, which is electrochemically detected at the electrode surface (Figure 2). The combination of PtNPs and GONS provided a high-surface-area conductive interface and a favorable environment for enzyme immobilization, contributing to enhanced electrochemical response.

The resulting biosensor exhibited excellent analytical performance, including a low limit of detection of 1 nM, a broad linear detection ranges from 0.001 to 200 μM, a rapid response time of approximately 3 s, and storage stability for up to six months without significant signal deterioration. These characteristics demonstrate the ability of the PtNP/GONS nanostructured interface to improve the analytical performance of the dual-enzyme sensing system while maintaining operational stability over an extended storage period.

Importantly, unlike the majority of previously reported ACh biosensors, which had only been evaluated in standard solutions or artificially spiked samples, the device was successfully validated using serum samples obtained from patients with Alzheimer’s disease and healthy controls. Specifically, serum samples from 20 patients with Alzheimer’s disease and 20 apparently healthy individuals were analyzed using the biosensor. The measured serum ACh concentrations were markedly lower in the Alzheimer’s disease group, with reported values ranging approximately from 1.0 to 6.5 nM, compared with approximately 9.0 to 12.0 nM in the healthy group. Furthermore, ACh concentrations obtained with the biosensor showed a strong correlation with those determined using a standard enzymatic colorimetric method (R^2^ = 0.989), providing evidence of good agreement between the proposed electrochemical platform and the reference method. The lower ACh levels observed in the Alzheimer’s disease group are consistent with the cholinergic dysfunction associated with the disease and highlight the potential of serum ACh measurement for distinguishing between the investigated groups. However, these findings should not be interpreted as establishing ACh as an independent biomarker of cognitive decline, since the study involved a relatively limited cohort and did not establish diagnostic or prognostic performance.

Collectively, these findings underscore the clinical relevance of this platform and mark an important milestone in the translation of ACh biosensing towards point-of-care diagnostic applications for neurodegenerative diseases [22]. The excellent analytical performance of this platform can be largely attributed to the synergistic combination of graphene oxide and platinum nanoparticles, which increases the electroactive surface area, facilitates electron transfer, and provides a favorable microenvironment for efficient enzyme immobilization. Nevertheless, the use of a dual-enzyme recognition system inevitably increases fabrication complexity and may compromise batch-to-batch reproducibility and long-term manufacturing scalability compared with non-enzymatic sensing strategies. From a translational perspective, however, its successful validation using serum samples from patients with Alzheimer’s disease represents a significant advance over most ACh biosensors, which remain limited to proof-of-concept studies performed in standard or artificially spiked samples. Consequently, this work establishes an important benchmark for the future clinical development of electrochemical biosensors.

Beyond enzyme-based amperometric platforms, recent research has also explored alternative electrochemical transduction mechanisms aimed at simplifying sensor architectures, reducing the dependence on enzymatic recognition, and improving applicability in complex biological environments. Particular attention should be given to the development of a label-free impedimetric biosensor based on electrochemical impedance spectroscopy (EIS), which represents a significant advance in ACh sensing strategies [72]. The approach is particularly relevant because it combines reagent-free, label-free detection with validation in biologically complex matrices, including rat blood and brain tissue, addressing an important limitation of many ACh biosensors that have primarily been evaluated in standard buffer solutions or artificially spiked samples. This demonstration therefore provides evidence for the feasibility of impedance-based ACh sensing under biologically relevant conditions and highlights its potential for neurochemical monitoring.

The biosensor consisted of a gold electrode functionalized through covalent immobilization of AChE using dithiobis (succinimidyl propionate) (DSP) as a crosslinking agent. Rather than detecting an electroactive product generated by the enzymatic reaction, as in conventional amperometric ACh biosensors, the system monitored changes in interfacial impedance resulting from the interaction between ACh and the immobilized AChE. This transduction mechanism enabled reagent-free and label-free measurements while maintaining the enzymatic recognition of ACh.

The sensor exhibited a linear response over the concentration range of 5.5–550 μM and was successfully evaluated in rat blood and ex vivo brain tissue. Importantly, the authors also investigated the device following implantation into rat brain tissue, where stable sensor performance was maintained for approximately four hours. These results demonstrate the feasibility of impedimetric detection of ACh in biologically relevant neural environments and support the potential of this approach for future implantable neurochemical monitoring systems.

Compared with conventional amperometric ACh biosensors, this label-free impedimetric strategy represents a conceptually different sensing approach by eliminating the need for electrochemical detection of enzymatically generated products such as hydrogen peroxide. This simplification reduces reagent dependence and may facilitate the development of implantable sensing devices with lower operational complexity. However, impedance-based measurements are intrinsically more sensitive to variations in the electrode interface, including nonspecific adsorption, biofouling, and changes in surface chemistry, which may compromise long-term analytical reproducibility under physiological conditions. Consequently, although this work constitutes a major step toward continuous in vivo monitoring, further optimization of surface engineering and antifouling strategies will be essential before clinical translation can be realistically achieved.

Beyond neurodegenerative diseases, recent studies have also demonstrated the potential of ACh biosensors in other clinically relevant applications. An example of the clinical translation of portable ACh biosensing is the development of an electrochemical sensor for the intraoperative identification of ganglionic regions in patients with Hirschsprung disease [73]. The device employed an anodized laser-induced graphene-modified screen-printed electrode functionalized with AChE and ChOx, enabling rapid determination of neurotransmitter levels directly in tissue biopsies (Figure 3). This system illustrates the potential of portable electrochemical platforms for real-time measurements in clinically relevant settings.

Unlike most previously reported ACh biosensors, which were validated using standard solutions or artificially spiked samples, this platform was evaluated using human biopsy specimens collected during surgery. The sensor provided objective and rapid neurochemical information that could support surgical decision-making and improve the identification of transition zones during resection procedures.

Although the clinical application addressed by this study differs from neurodegenerative disorders, the successful implementation of a portable biosensor in a real clinical setting highlights the translational potential of electrochemical ACh sensing technologies. The successful validation of ACh biosensors in human biological samples highlights their translational potential and demonstrates the feasibility of incorporating ACh monitoring into routine clinical workflows [22,73]. From a translational perspective, this study marks an important step in the evolution of biosensing, extending the focus beyond analytical performance towards real-world clinical application [73]. Unlike many recently reported platforms that primarily emphasize progressively lower detection limits, this study demonstrates that rapid response, portability, ease of operation, and integration into routine clinical workflows may ultimately represent more decisive factors for successful medical adoption. Nevertheless, broader multicenter validation studies involving larger patient cohorts will still be required to establish the robustness, reproducibility, and clinical utility of this technology before widespread implementation can be considered.

Collectively, enzymatic electrochemical biosensors currently remain the most analytically mature strategy for ACh detection, combining excellent sensitivity with extensive experimental validation. However, their dependence on enzyme immobilization and catalytic activity continues to limit long-term operational stability, motivating the development of alternative sensing concepts capable of maintaining comparable analytical performance while improving robustness and ease of fabrication.

#### 4.2.2. Non-Enzymatic Electrochemical Sensors

The emergence of non-enzymatic ACh sensors represent one of the most significant conceptual advances in recent electrochemical biosensing. Rather than pursuing incremental improvements in conventional enzyme-based architecture, these platforms seek to overcome fundamental limitations associated with enzyme instability, limited shelf life, and complex immobilization procedures. Consequently, recent research has increasingly focused on engineering catalytic nanomaterials capable of combining high analytical sensitivity with enhanced operational robustness and manufacturing simplicity.

In contrast to enzymatic devices, non-enzymatic electrochemical sensors rely on electrocatalytic materials capable of directly interacting with ACh without incorporating biological recognition elements. These systems generally exhibit greater operational stability, lower manufacturing costs, and improved shelf life, since they avoid the intrinsic limitations associated with enzymes, including denaturation, loss of activity during storage, and sensitivity to environmental conditions such as pH and temperature [14,22]. However, the absence of highly selective molecular recognition mechanisms often results in lower selectivity and increased susceptibility to interference from electroactive compounds present in complex biological matrices.

Recent developments in nanomaterials have considerably improved the analytical performance of non-enzymatic ACh sensors by enhancing electron transfer kinetics and increasing the number of catalytically active sites. Among these approaches, an enzyme-free electrochemical sensor based on carbon-coated copper microspheres deposited onto a carbon electrode was developed [71]. The combination of conductive carbon layers with electrocatalytically active copper microspheres provided a highly porous surface with a large electrochemically active area, enabling sensitive and selective determination of ACh in human serum samples spiked with the analyte. The sensor exhibited a linear response between 10 and 800 μM with a detection limit of 100 nM, demonstrating that enzyme-free approaches can achieve adequate analytical performance while substantially simplifying sensor fabrication and reducing production costs.

An alternative approach was based on the development of an enzyme-free sensor comprising vanadium pentoxide nanorods (V_2_O_5_ NRs) dispersed within a Nafion^®^ matrix and immobilized on a glassy carbon electrode [69]. The V_2_O_5_ nanorods acted as highly efficient electrocatalytic centers, while Nafion^®^ contributed to improved film stability and reduced interference from negatively charged species. Remarkably, this device achieved a detection limit of 0.011 nM and a broad linear range extending from 0.0001 to 100 μM, representing one of the lowest limits of detection reported among the non-enzymatic electrochemical ACh sensors included in this review (Figure 4). This platform is presented as an illustrative example of the diverse non-enzymatic electrochemical strategies explored for ACh detection.

These findings highlight the potential of transition metal oxide nanostructures for ultrasensitive neurotransmitter detection. Although the analytical performance reported by Hussain et al. is remarkable, particularly in terms of detection limit, the practical significance of achieving picomolar-level sensitivity deserves careful consideration. Since physiological ACh concentrations in biological fluids generally lie within the nanomolar range, further reductions in the detection limit may provide only limited additional clinical benefit. Under these circumstances, improving selectivity, long-term operational stability, resistance to matrix effects, and reproducibility in complex biological samples may represent more relevant objectives than pursuing progressively lower analytical detection limits. Consequently, future development of non-enzymatic ACh sensors should increasingly prioritize robustness and clinical applicability alongside analytical sensitivity.

Once analytical performance reached physiologically relevant concentration ranges, research priorities gradually expanded beyond sensitivity toward device engineering, giving rise to flexible and implantable biosensors designed for continuous vivo monitoring. A particularly innovative non-enzymatic strategy was the introduction of the Flexible Acetylcholine Sensing Thread (FAST), representing the first flexible solid-contact potentiometric platform developed for real-time ACh monitoring in neural tissues [74]. Unlike conventional amperometric biosensors based on enzymatic conversion of ACh or electrocatalytic oxidation at modified electrode surfaces, FAST relies on direct potentiometric recognition of ACh using a calix [4] arene ionophore incorporated into an ion-selective membrane. The sensing platform was fabricated on a flexible cotton thread coated with conductive ink and sealed with a thin Parylene C layer, resulting in a probe with a diameter of approximately 400 μm suitable for minimally invasive applications. The sensor exhibited a near-Nernstian response with a linear range between 1 μM and 10 mM and a detection limit of 2.6 μM in deionized water, although the detection limit increased to 20 μM in artificial cerebrospinal fluid because of the high ionic background of the medium. Importantly, the device successfully quantified ACh in fresh sheep brain tissue and brain homogenates without sample pretreatment, demonstrating the feasibility of direct measurements in complex biological matrices. Although its analytical sensitivity remains lower than that of state-of-the-art enzymatic amperometric biosensors, the flexible architecture represents an important advance toward implantable electrochemical platforms for continuous monitoring of cholinergic signaling. The authors also identified several challenges that must be addressed before clinical translation, including improving long-term stability, minimizing water-layer formation at the solid-contact interface, and enhancing selectivity in complex neural environments through the development of more selective ionophores and optimized sensing membranes. These findings illustrate the current trend toward engineering electrochemical sensors that prioritize flexibility, biocompatibility, and long-term in vivo operation in addition to analytical performance.

The FAST platform also illustrates an important evolution in ACh sensor design, where analytical performance is no longer the sole criterion for technological advancement. Although its detection limit is less competitive than those reported for state-of-the-art enzymatic amperometric biosensors, its flexible architecture, mechanical compliance, and compatibility with neural tissues address key challenges associated with chronic implantation that conventional rigid electrodes cannot readily overcome. This shift highlights an emerging trend in neurochemical sensing, in which biocompatibility, mechanical stability, and long-term functionality are equally important performance indicators alongside sensitivity and detection limit. Consequently, future implantable ACh sensors will likely require a balanced optimization of analytical, mechanical, and biological properties rather than maximizing a single analytical parameter.

Overall, non-enzymatic electrochemical sensors represent an attractive alternative to conventional enzyme-based biosensors, particularly for applications requiring long-term stability and simplified manufacturing. Nevertheless, improving selectivity in complex biological matrices remains one of the major challenges limiting their clinical translation. In contrast, enzymatic electrochemical biosensors currently remain the most mature and clinically advanced technology for ACh quantification due to their superior specificity and compatibility with biological samples.

The studies summarized below illustrate how electrochemical ACh biosensors have progressively evolved from conventional dual-enzyme configurations toward more sophisticated platforms integrating nanomaterials, label-free sensing strategies, flexible electronics, and clinically relevant protocols of validation.

Overall, conventional analytical methods have provided valuable analytical performance for ACh determination and have played a fundamental role in the characterization of cholinergic neurotransmission. However, their limited portability, high instrumentation costs, extensive sample preparation, and limited suitability for continuous and real-time monitoring have stimulated the development of biosensor technologies [8,27]. By combining biological recognition elements with miniaturized transducers, biosensors offer rapid, sensitive, and potentially point-of-care detection of ACh, making them particularly attractive for neurochemical monitoring and applications related to neurological and neurodegenerative disorders [26,27].

In this context, the following section focuses on electrochemical and optical biosensor platforms for ACh detection, with particular emphasis on the technological strategies developed to improve sensitivity, selectivity, stability, miniaturization, and real-time monitoring capabilities. To facilitate comparison and enhance the pedagogical value of the review, the main analytical and technological characteristics of representative ACh biosensing platforms are summarized in Table 1 and Table 2, while selected figures illustrate representative sensing mechanisms and device configurations. These elements highlight the diversity of sensing strategies reported for ACh detection while maintaining the specific focus of this review.

Compared with enzymatic platforms, non-enzymatic electrochemical sensors offer a clear advantage in terms of operational stability and simplified device architecture by eliminating biological recognition elements. Nevertheless, achieving the same degree of selectivity in complex biological matrices remains one of their principal challenges, indicating that both sensing strategies currently provide complementary rather than mutually exclusive solutions.

Collectively, recent advances in electrochemical ACh biosensors reveal a clear shift in research priorities. While early studies largely focused on maximizing analytical sensitivity through increasingly sophisticated electrode modifications, more recent developments increasingly emphasize clinical applicability, long-term operational stability, and compatibility with physiologically relevant environments. This evolution is reflected by the emergence of flexible implantable platforms, label-free impedimetric strategies, and non-enzymatic sensing concepts designed to overcome the intrinsic limitations associated with enzyme stability and conventional electrode architectures. Importantly, the analytical performance of many recently reported electrochemical biosensors has already reached concentration ranges well below physiological ACh levels, suggesting that further improvements in detection limits alone are unlikely to substantially advance the field. Instead, future progress will largely depend on enhancing analytical selectivity in complex biological matrices, minimizing biofouling, improving manufacturing reproducibility, and, most importantly, achieving extensive validation in authentic clinical samples. Consequently, the next generation of electrochemical ACh biosensors will likely be distinguished not by achieving ever-lower detection limits, but by demonstrating robust performance in real clinical settings, long-term operational stability, scalable manufacturing, and clear clinical utility.

### 4.3. Optical Biosensors

In parallel with advances in electrochemical sensing, optical biosensors followed a largely independent evolutionary pathway driven by the need to achieve high-resolution imaging, multiplexed detection, and non-contact monitoring rather than simply improving analytical sensitivity.

Furthermore, optical transduction mechanisms can be readily integrated into miniaturized platforms, optical fibers, and implantable devices, making them particularly attractive for future biomedical and neurochemical monitoring applications [14]. The main optical biosensing approaches reported for acetylcholine determination, together with their analytical characteristics and applications, are summarized in Table 2.

The rapid development of optical ACh biosensors reflect the growing demand for sensing technologies capable of providing information beyond simple analyte quantification. In contrast to electrochemical approaches, recent optical platforms increasingly exploit fluorescence, plasmonics, and photonic nanostructures to enable multiplexed measurements, high spatial resolution, and compatibility with in vivo imaging. Consequently, the evolution of optical biosensing has been driven not only by improvements in analytical sensitivity but also by the need to visualize cholinergic signaling within increasingly complex biological systems.

Like electrochemical systems, optical sensors for ACh detection can be broadly classified into enzymatic and non-enzymatic approaches. However, most optical platforms reported to date rely on enzymatic recognition based on AChE-mediated hydrolysis reactions, which generate either optical or chemical intermediates that can subsequently be converted into measurable optical signals [14,22].

An important trend emerging from recent studies concerns the biological matrices employed for validation. Most optical sensors have been evaluated exclusively in standard ACh solutions or artificially enriched samples for example, validation of the liquid crystal sensor and the fibre-optic surface plasmon resonance (SPR) platform was performed exclusively using standard ACh solutions [75,76], whereas successful operation in spiked human serum samples was also demonstrated in other studies [77,78]. Similarly, validation of the nanozyme-based colorimetric platform was performed using enriched human serum samples [79]. To date, however, no optical biosensor has been validated using samples from patients with neurodegenerative diseases, highlighting an important translational gap compared with recent electrochemical approaches.

Although optical and electrochemical biosensors are frequently presented as alternative technologies, their respective strengths suggest that they are better regarded as complementary analytical platforms. Whereas electrochemical sensors currently provide greater potential for portable and point-of-care applications, optical approaches uniquely enable high-resolution imaging and spatially resolved monitoring of cholinergic activity.

#### 4.3.1. Colorimetric Biosensors

Colorimetric biosensors represent one of the simplest and most accessible optical sensing approaches. These devices typically rely on AChE-mediated hydrolysis of Ach followed by chromogenic reactions or nanozyme-catalyzed oxidation processes that produce visible color changes proportional to analyte concentration. Their simplicity, low cost, and minimal instrumentation requirements make them particularly attractive for point-of-care applications and resource-limited settings.

Recent developments have focused on the incorporation of nanozymes and nanostructured materials to improve analytical sensitivity and long-term stability. A colorimetric biosensor was developed based on montmorillonite nanosheets decorated with Co_0.5_Ni nanoparticles, which acted as peroxidase mimetics capable of catalyzing hydrogen peroxide decomposition following enzymatic ACh hydrolysis. The sensor achieved a detection limit of 565 nM and demonstrated successful operation in spiked human serum samples [77].

Similarly, a bifunctional enzyme-mimicking metal–organic framework (MOF) incorporating platinum nanoparticles as catalytic centers was reported [79]. Unlike conventional enzymatic systems, this platform exploited nanozyme activity to improve stability and reduce enzyme-related limitations. Although the system exhibited a higher detection limit of approximately 5 μM, it demonstrated excellent robustness and compatibility with serum matrices, highlighting the potential of MOF-based optical sensing platforms for future biosensor development.

#### 4.3.2. Fluorescent Biosensors

Fluorescence-based biosensors generally provide higher sensitivity than colorimetric systems owing to their superior signal-to-noise ratios and the availability of efficient signal amplification mechanisms. Recent advances in nanotechnology have driven the development of fluorescent probes based on carbon quantum dots, metallic nanoclusters, and semiconductor nanomaterials for ultrasensitive neurotransmitter detection.

Among the optical sensing platforms reported in the literature, the optical fibre-based approach stands out as one of the most sensitive systems developed for ACh determination [78]. The device employed pH-responsive fluorescent carbon quantum dots immobilized within cellulose acetate matrices deposited onto optical fibers. The enzymatic hydrolysis of ACh generated local pH changes that modulated fluorescence intensity, enabling indirect quantification of the neurotransmitter.

The sensor achieved an impressive detection limit of 0.29 nM and a linear detection range between 10^−3^ and 0.1 μM, values comparable to those obtained using state-of-the-art electrochemical biosensors. Beyond conventional fluorescent biosensors, genetically encoded probes have emerged as powerful tools for monitoring cholinergic signaling in living systems. Jing et al. developed the optimized GRAB-ACh3.0 sensor, which combines high specificity with rapid response kinetics (τon ≈ 105 ms and τoff ≈ 3.7 s). The sensor enabled real-time visualization of acetylcholine dynamics in multiple animal models and represents a significant advance for studying cholinergic neurotransmission with high spatiotemporal resolution [80].

Furthermore, successful operations in human serum samples enriched with ACh demonstrated the feasibility of combining fluorescence sensing with clinically relevant biological matrices. The outstanding sensitivity achieved by this fluorescence-based platform primarily arises from the inherently high signal-to-noise ratio of optical fluorescence measurements together with the excellent photophysical properties of carbon quantum dots. Nevertheless, fluorescence biosensors generally require relatively sophisticated optical instrumentation, careful control of excitation and emission conditions, and may be affected by photobleaching, background autofluorescence, and variations in sample optical properties. Consequently, despite their remarkable analytical performance, the widespread clinical implementation of fluorescence based ACh biosensors will likely depend on further advances in portable optical instrumentation and standardized measurement protocols capable of ensuring robust performance outside controlled laboratory environments.

#### 4.3.3. Surface Plasmon Resonance Biosensors

Surface plasmon resonance (SPR)-based biosensors enable label-free monitoring of molecular interactions by measuring changes in the refractive index occurring near metallic surfaces. Because signal generation does not require electroactive species or fluorescent labels, SPR platforms are particularly attractive for continuous and non-destructive measurements.

Recent efforts have focused on combining plasmonic nanostructures with optical fiber technologies to improve sensitivity while reducing device size. A representative example is a fiber-optic SPR biosensor based on resonance coupling between gold nanorods and thin gold films [76]. The incorporation of gold nanorods significantly enhanced local electromagnetic fields and improved plasmonic sensitivity, enabling ACh quantification over the concentration range of 3.42–34.2 μM.

Although the detection limit of 3.08 μM remains higher than those achieved by electrochemical platforms, the study demonstrated the feasibility of miniaturized fiber-optic SPR devices for neurotransmitter detection and highlighted their potential for integration into implantable or minimally invasive monitoring systems.

To further improve sensitivity, a localized surface plasmon resonance (LSPR)-based reflective fiber-optic biosensor incorporating gold nanorods as signal-enhancing nanostructures was proposed [81]. The biosensor achieved an ultralow detection limit of 3.9 × 10^−11^ M and a wide linear range extending from 10^−10^ to 10^−4^ M. These results highlight the potential of nanoplasmonic platforms for highly sensitive acetylcholine detection in complex analytical applications. Temperature-related signal fluctuations remain an important challenge in fiber-optic biosensing. To address this issue, a temperature-compensated U-shaped fiber-optic biosensor was developed by immobilizing acetylcholinesterase onto a gold-coated sensing region. The device provided selective acetylcholine detection with a detection limit of 30 nM while incorporating temperature compensation capabilities that improved measurement reliability. Such strategies are particularly attractive for future point-of-care and real-time sensing applications [82].

Overall, optical biosensors currently lag behind electrochemical systems in terms of analytical sensitivity and clinical validation. Nevertheless, their intrinsic advantages, including immunity to electrical noise, compatibility with multiplexed measurements, and suitability for remote and minimally invasive sensing, position them as highly promising technologies for future neurochemical monitoring applications, particularly when combined with optical fibers, nanophotonic structures, and implantable imaging platforms.

Unlike electrochemical platforms, the recent evolution of optical ACh biosensors have primarily focused on diversifying transduction mechanisms rather than fundamentally changing the underlying biorecognition strategy, resulting in complementary analytical capabilities adapted to different biomedical applications.

**Table 2:** Optical sensors for ACh determination described in recent studies (2020–2026).

Study	Transductor	Detection Technique	Recognition Element	Immobilization/ Modification Strategy	Sample	Linear Range (LOD)	Assay/Response Time
[75]	LC	POM	AChE	Myr SAM	Standard aqueous ACh solutions	0.01–1 ×10^3^ μM (14.5 nM)	1 h
[77]	Co_0.5_Ni_0.5_Fe_2_O_4_-decorated MMT	Colorimetric	AChE	Co_0.5_Ni_0.5_Fe_2_O_4_-MMT nanozyme	NR	0.1–30 µM (57 nM)	NR
[78]	Optical fiber modified with N-CQDs	Fluorescence	AChE	N-CQDs (cross-linking)	Spiked serum	1–100 nM (0.29 nM); 20–200 μM (16.28 μM)	10 h
[76]	Gold film/coated optical fiber	SPR	AChE	GNRs (cross-linking)	NR	0.5–5 μg mL^−1^ (0.45 μg mL^−1^)	NR
[79]	MOF-808/Pt NPs nanozyme	Colorimetric	-	MOF-808/Pt NPs bifunctional nanozyme	Spiked human serum	33–6700 μM (5 µM)	1.7 h
[81]	GNRs-modified reflective fiber-optic sensor	LSPR	AChE	MUA-EDC/NHS (covalent)	ACh standard solutions	10^−10^–10^−4^ M (0.039 nM)	12 h
[80]	GRAB-ACh3.0 genetically encoded sensor	Fluorescence imaging	Engineered muscarinic acetylcholine receptor (M3R)	Not applicable—genetically encoded sensor	HEK293T cells (in vitro); mouse brain and Drosophila olfactory system (in vivo)	NR	NR
[82]	U-shaped SMF-NCF-SMF fiber sensor with Au coating	SPR + MMI	AChE	MUA-EDC/NHS (covalent)	ACh standard solutions	NR (30 nM)	≈46 min (signal stabilization)
[57]	D-shaped photonic crystal fiber (PCF)	SPR	ACh	D-shaped PCF with dual-layer Au/TiO_2_ plasmonic coating; ANN-based refractive index (RI) prediction	ACh standard solutions	NR wavelength sensitivity up to 34,442.9571 nm RIU^−1^; amplitude sensitivity 72.5422 RIU^−1^	NR
[64]	Optical fiber	SPR	AChE	Ti_3_C_2_ MXene/GNRs assembled by electrostatic layer-by-layer deposition;	Spiked milk	0–700 µM (4.42 μM)	220 s (signal)
[62]	Optical fiber with GNRs/Ti_3_C_2_ MXene	LSPR	AChE	Electrostatic self-assembly of Ti_3_C_2_ MXene nanosheets onto GNR-based optical fiber probe	ACh standard solutions	0–900 μM (6.58 μM)	NR

Abbreviations: ACh: acetylcholine; AChE: acetylcholinesterase; ANN: artificial neural network; EDC: 1-ethyl-3-(3-dimethylaminopropyl)carbodiimide; GNRs: gold nanorods; LC: liquid crystal; LOD: limit of detection; LSPR: localized surface plasmon resonance; MMI: multimode interference; MMT: montmorillonite; MOF: metal–organic framework; MUA: 11-mercaptoundecanoic acid; Myr: myristoylcholine; N-CQDs: N-doped carbon quantum dots; NCF: no-core fiber; NHS: N-hydroxysuccinimide; POM: polarized optical microscopy; PCF: photonic crystal fiber; Pt NPs: platinum nanoparticles; RI: refractive index; RIU: refractive index unit; NR: not reported; SAM: self-assembled monolayer; SMF: single-mode fiber; SPR: surface plasmon resonance.

Collectively, recent advances in optical ACh biosensors indicate that these platforms should not be viewed as direct competitors to electrochemical sensors, but rather as complementary technologies addressing different analytical and biomedical needs. Whereas electrochemical biosensors currently offer superior clinical maturity, simpler instrumentation, and greater compatibility with portable point-of-care devices, optical platforms provide unique capabilities for non-contact measurements, multiplexed detection, real-time imaging, and high spatial resolution that cannot be readily achieved using electrochemical approaches. Among the various optical strategies, fluorescence-based biosensors currently demonstrate the highest analytical sensitivity, while SPR platforms offer label-free monitoring of biomolecular interactions and colorimetric systems remain attractive for low-cost and resource-limited applications. Nevertheless, despite their remarkable analytical capabilities, most optical biosensors have been validated only in standard solutions or artificially enriched samples, highlighting a substantial gap between laboratory proof-of-concept studies and clinical implementation. Consequently, future progress in optical ACh sensing will likely depend less on further improvements in analytical sensitivity than on the development of portable optical instrumentation, standardized analytical protocols, and extensive validation in authentic biological samples. This observation suggests that the current limitations of optical ACh biosensors are no longer primarily determined by optical transduction mechanisms, but rather by the lack of clinically validated sensing platforms capable of operating reliably in complex physiological environments. Rather than replacing electrochemical biosensors, optical sensing technologies are expected to play an increasingly complementary role within multimodal diagnostic platforms that combine the strengths of both approaches for comprehensive neurochemical monitoring.

### 4.4. Emerging Optical Molecular Sensors for in Vivo ACh Imaging

The convergence between optical biosensors and neuroimaging technologies has recently given rise to genetically encoded fluorescent probes and nanosensors capable of monitoring ACh dynamics directly in living organisms, representing an increasingly important area of cholinergic sensing research. Beyond conventional biosensors, genetically encoded fluorescent sensors have transformed the study of cholinergic neurotransmission by enabling real-time monitoring of ACh dynamics with high temporal and spatial resolution [7,83]. Although these systems are not biosensors in the traditional analytical sense, they represent highly valuable molecular sensing tools for investigating cholinergic function in physiological and pathological conditions.

Among the most widely used platforms are the GPCR Activation-Based ACh sensors (GRAB-ACh) and the intensity-based ACh sensing fluorescent reporter (iAChSnFR). GRAB-ACh sensors are based on engineered muscarinic M3 receptors fused to circularly permuted green fluorescent proteins (cpGFPs). Binding of ACh induces conformational changes in the receptor that are translated into variations in fluorescence intensity, allowing direct visualization of cholinergic activity with sub-second temporal resolution [84]. Subsequent optimization of GRAB-ACh sensors further improved their sensitivity and kinetics, enabling the detection of ACh dynamics in vivo using approaches including fiber photometry and two-photon imaging [80].

In contrast, iAChSnFR is based on a bacterial periplasmic ACh-binding protein coupled to a conformationally sensitive fluorescent protein. These sensors exhibit rapid kinetics and high specificity while avoiding direct activation of endogenous muscarinic receptors, making them particularly suitable for quantitative measurements of extracellular ACh dynamics. Together with GRAB-ACh sensors, iAChSnFR illustrates the development of genetically encoded optical probes based on distinct molecular recognition strategies for monitoring extracellular ACh.

These genetically encoded indicators are commonly combined with in vivo fiber photometry or two-photon microscopy, enabling monitoring of cholinergic neurotransmission in behaving animals and, depending on the imaging approach, at cellular or circuit level resolution. Such approaches have provided important insights into the role of ACh in attention, learning, reward processing, and memory formation, as well as into alterations of cholinergic signaling associated with neurological and neurodegenerative disorders [7,80].

In addition to genetically encoded probes, synthetic fluorescent nanosensors have also emerged as promising alternatives for in vivo ACh imaging. A fluorescent nanosensor incorporating AChE was developed and a DNA-based sensing architecture capable of monitoring ACh release in the PNS in vivo [85]. The sensor enabled detection and spatial mapping of endogenous ACh release in living mice, demonstrating the feasibility of optical approaches for real-time neurochemical imaging outside the CNS [85].

Despite their remarkable capabilities, genetically encoded sensors currently remain largely restricted to preclinical neuroscience research because they require viral delivery systems or transgenic expression strategies. Their application also depends on optical access and specialized fluorescence imaging instrumentation, which currently limits their direct translation to routine clinical applications. Nevertheless, their ability to provide dynamic measurements of cholinergic neurotransmission makes them invaluable tools for investigating neurodegenerative mechanisms and establishing relationships between ACh dynamics, neuronal activity, and behavior [7,83].

The evolution of genetically encoded optical probes further expands the concept of ACh biosensing by shifting the primary objective from quantitative detection toward direct visualization of neurotransmission. Consequently, these technologies should be regarded as complementary tools for fundamental neuroscience and mechanistic studies of cholinergic signaling, rather than direct substitutes for conventional diagnostic biosensors. Their principal advantage lies in their ability to resolve the spatial and temporal dynamics of ACh signaling, whereas conventional electrochemical biosensors are generally more readily adapted to quantitative chemical analysis and miniaturized sensing platforms.

The rapid diversification of ACh biosensing strategies has resulted in platforms with distinct analytical capabilities, technological maturity, and potential biomedical applications. Taken together, the electrochemical and optical approaches discussed above illustrate that current ACh sensing technologies are characterized by complementary strengths and limitations rather than by a single universally superior sensing strategy.

As shown in Table 3, no single biosensing platform simultaneously maximizes sensitivity, selectivity, operational stability, portability, implantability, and clinical maturity. Instead, the field is progressively evolving toward complementary technological solutions, in which the selection of a particular sensing strategy should be guided by the specific analytical objective rather than by analytical sensitivity alone. This trend reflects the increasing maturity of ACh biosensor research and the transition from technology-driven development toward application-oriented design.

This comparison also highlights an important transition in the field: analytical sensitivity alone is becoming an insufficient criterion for evaluating the technological maturity of ACh biosensors. Increasing attention is instead being directed toward robustness, compatibility with complex biological environments, miniaturization, continuous monitoring, and validation in clinically relevant samples.

## 5. Current Challenges and Future Perspectives

The rapid evolution of ACh biosensors over the past decade has substantially improved several analytical parameters that initially limited neurotransmitter detection. Recent platforms have achieved low detection limits, improved selectivity, and enhanced analytical performance using nanostructured materials, advanced electrode architectures, and alternative recognition strategies. However, high analytical sensitivity alone does not ensure successful biomedical translation. The main challenges now concern reliable operation in complex biological environments, long-term stability, reproducible fabrication, biocompatibility, miniaturization, and validation under clinically relevant conditions. Thus, research is gradually shifting from proof-of-concept demonstrations toward sensing systems capable of maintaining reliable performance under realistic biological conditions [14,26,86].

No single ACh sensing technology currently meets all analytical and biomedical requirements. Enzymatic electrochemical biosensors provide high specificity and relatively simple signal transduction, whereas non-enzymatic platforms can avoid some of the stability limitations associated with biological recognition elements. Optical approaches offer complementary advantages, particularly for spatially resolved measurements, while nanomaterials can improve signal transduction and facilitate miniaturization. The most promising developments will therefore likely involve combining these approaches according to the requirements of the intended application [8,24,87].

One of the main limitations is the restricted use of physiologically and clinically representative samples during sensor validation. Many reported ACh sensors are still evaluated using standard solutions or artificially supplemented biological matrices. Although these experiments are useful for determining sensitivity, selectivity, linear range, and recovery, they do not reproduce the complexity of authentic biological environments, where proteins, lipids, salts, metabolites, cells, and related compounds can affect mass transport, electrode reactions, enzymatic activity, and signal stability. Different studies have demonstrated ACh detection in human biological samples represent important steps toward translational application [22,73]. Nevertheless, detection in human samples should not be equated with clinical validation, which requires larger patient cohorts, comparison with established analytical methods, and evaluation across relevant pathological conditions.

Biofouling and the resulting loss of sensor performance are further concerns for prolonged operation in biological fluids. Adsorption of proteins and other biomolecules onto the sensing interface can block active sites, alter mass transport and charge-transfer processes, and cause signal attenuation or drift. These effects are particularly relevant to implantable and wearable sensors designed for continuous monitoring. A recent work has shown that biomimetic interfacial strategies can improve the stability of biochemical sensors during in vivo operation, emphasizing the importance of controlling the biological interface in addition to optimizing the sensing material [88]. Future studies should therefore consider antifouling coatings, biomimetic interfaces, and biocompatible surface modifications to preserve sensor performance over extended periods [87].

For enzymatic ACh biosensors, the stability of the recognition element remains another important limitation. AChE and ChOx can be affected by pH, temperature, ionic strength, immobilization conditions, and storage, potentially resulting in loss of activity and signal deterioration. Although enzyme immobilization and nanostructured support can improve stability, they do not eliminate all these limitations. Alternative catalytic and recognition strategies, including nanozymes, biomimetic materials, metal–organic frameworks, and enzyme-free interfaces, are therefore attracting increasing attention. Recent studies demonstrate the potential of enzyme-mimicking materials for ACh analysis and suggest possible routes toward more stable sensing systems [69,71,79,89].

The reproducibility and scalability of nanomaterial-based biosensors also remain important barriers to translation. Graphene derivatives, metal nanoparticles, carbon-based materials, quantum dots, and metal–organic frameworks can exhibit variations in morphology and surface properties depending on synthesis and processing conditions. Such batch-to-batch differences may affect catalytic activity, charge-transfer kinetics, and analytical response. Standardized synthesis procedures, appropriate physicochemical characterization, quality-control protocols, and reproducible electrode fabrication will therefore be necessary to improve inter-device consistency and support future regulatory assessment [23,67].

Most current ACh biosensors are also designed for single-analyte detection. This is a limitation because neurodegenerative disorders involve changes in several neurotransmitter systems and metabolic pathways rather than cholinergic dysfunction alone. Simultaneous monitoring of ACh with dopamine, glutamate, γ-aminobutyric acid (GABA), serotonin, or inflammatory biomarkers could provide a more comprehensive representation of disease-associated biochemical changes. Multiplexed platforms may therefore be useful for identifying neurochemical patterns rather than relying on a single biomarker. Recent advances in electrochemical biosensing have also expanded towards emerging biomarkers such as exosomes, highlighting the potential of multimodal diagnostic platforms beyond single-analyte detection [90]. However, cross-reactivity, sensor-to-sensor interference, calibration, and reliable simultaneous detection remain significant challenges [7,87].

The integration of electrochemical biosensors with microfluidic technologies represents an important strategy for ACh monitoring. Microfluidic platforms enable precise manipulation of small sample volumes, controlled mass transport, reduced reagent consumption, and integration of multiple analytical steps within miniaturized devices. A dual-electrode amperometric microfluidic biosensor was reported in which sequential enzymatic reactions were spatially separated within a microfluidic channel [91]. The system achieved a detection limit of approximately 0.6 nM and was applied to human plasma and to Ca^2+^-induced ACh release from leukemic T cells, demonstrating the potential of microfluidic electrochemical sensing for cell-based neurotransmitter measurements in complex biological samples [91].

Beyond this specific implementation, the integration of sample handling, analyte transport, biochemical recognition, and electrochemical detection within lab-on-a-chip architectures could reduce sample requirements and facilitate the development of portable analytical systems. This combination is particularly attractive for decentralized measurements and point-of-care applications, where conventional laboratory-based procedures may be impractical. However, translating these platforms toward reliable biological measurements requires reproducible integration of microfluidic and sensing components, as well as robust performance in complex biological matrices. These challenges highlight the need for further optimization of microfluidic architectures, surface chemistry, and sensor integration before such systems can achieve broader practical and clinical applicability [54,91].

Wearable and implantable platforms offer another potential route toward continuous neurochemical monitoring. Flexible electrodes, microneedle arrays, soft bioelectronics, and miniaturized wireless sensors could enable repeated measurements while reducing the invasiveness of conventional sampling approaches. Recent progress in non-invasive electrochemical biosensing highlights the increasing relevance of wearable technologies for continuous health monitoring [92]. For ACh, these systems could provide information on dynamic cholinergic activity and treatment responses that cannot be obtained from single-time-point measurements. Their clinical use, however, will depend on overcoming challenges related to biocompatibility, mechanical stability, calibration drift, power supply, wireless communication, and reliable operation in vivo [85,87,88].

Artificial intelligence (AI) and advanced data-processing methods may also become useful as sensing platforms generate increasingly complex and multidimensional datasets. Machine-learning approaches could assist with signal interpretation, correction of baseline variations and matrix effects, and identification of patterns associated with some disease states. However, their application will require sufficiently large and representative datasets, together with independent validation, to ensure that model performance is robust across patients and devices.

Overall, future advances in ACh sensing will depend less on further improvements in detection limits alone and more on achieving reliable performance in realistic biological environments. Progress in nanomaterials, biomimetic interfaces, microfluidics, miniaturized electrochemical and optical transduction, and wireless technologies provide a strong foundation for this transition. Nevertheless, long-term in vivo validation, reproducible manufacturing, standardized benchmarking, and clinically meaningful validation remain essential before ACh biosensors can move from experimental platforms toward routine biomedical applications. If these challenges are addressed, ACh biosensors could become valuable tools for monitoring cholinergic dysfunction and for investigating dynamic neurotransmission in health and disease.

## Figures and Tables

**Figure 1:**
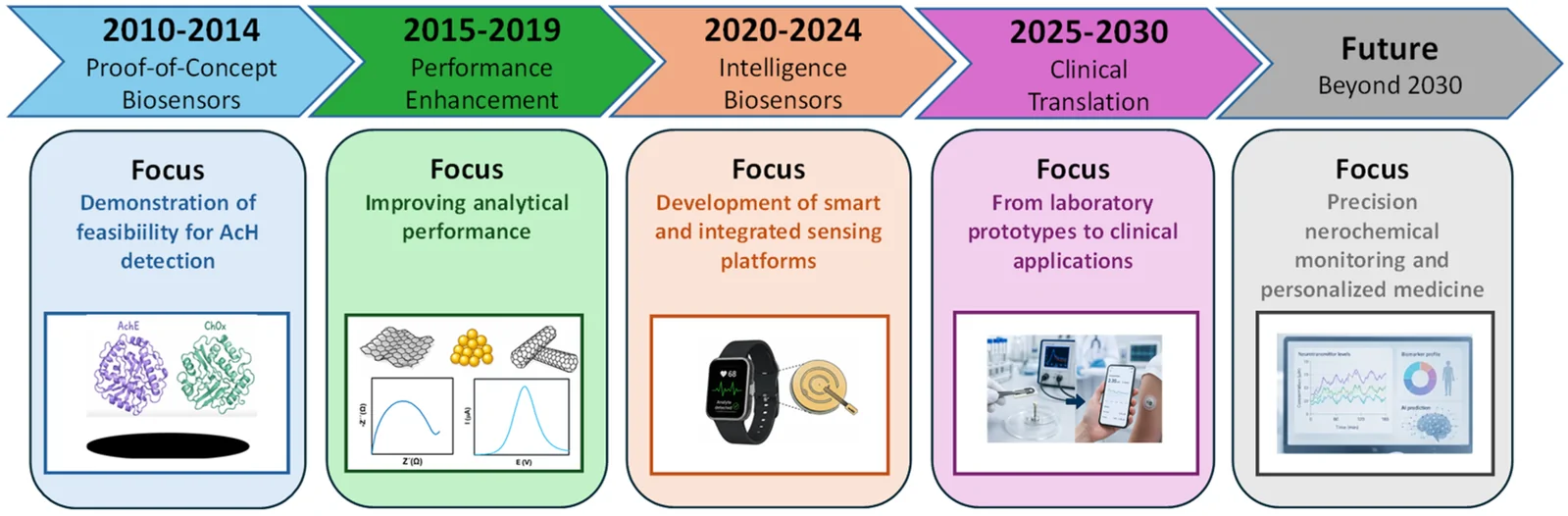
Schematic overview of the evolution of ACh biosensor technologies, from conventional enzyme-based sensors to advanced wearable and implantable platforms for clinical applications.

**Figure 2:**
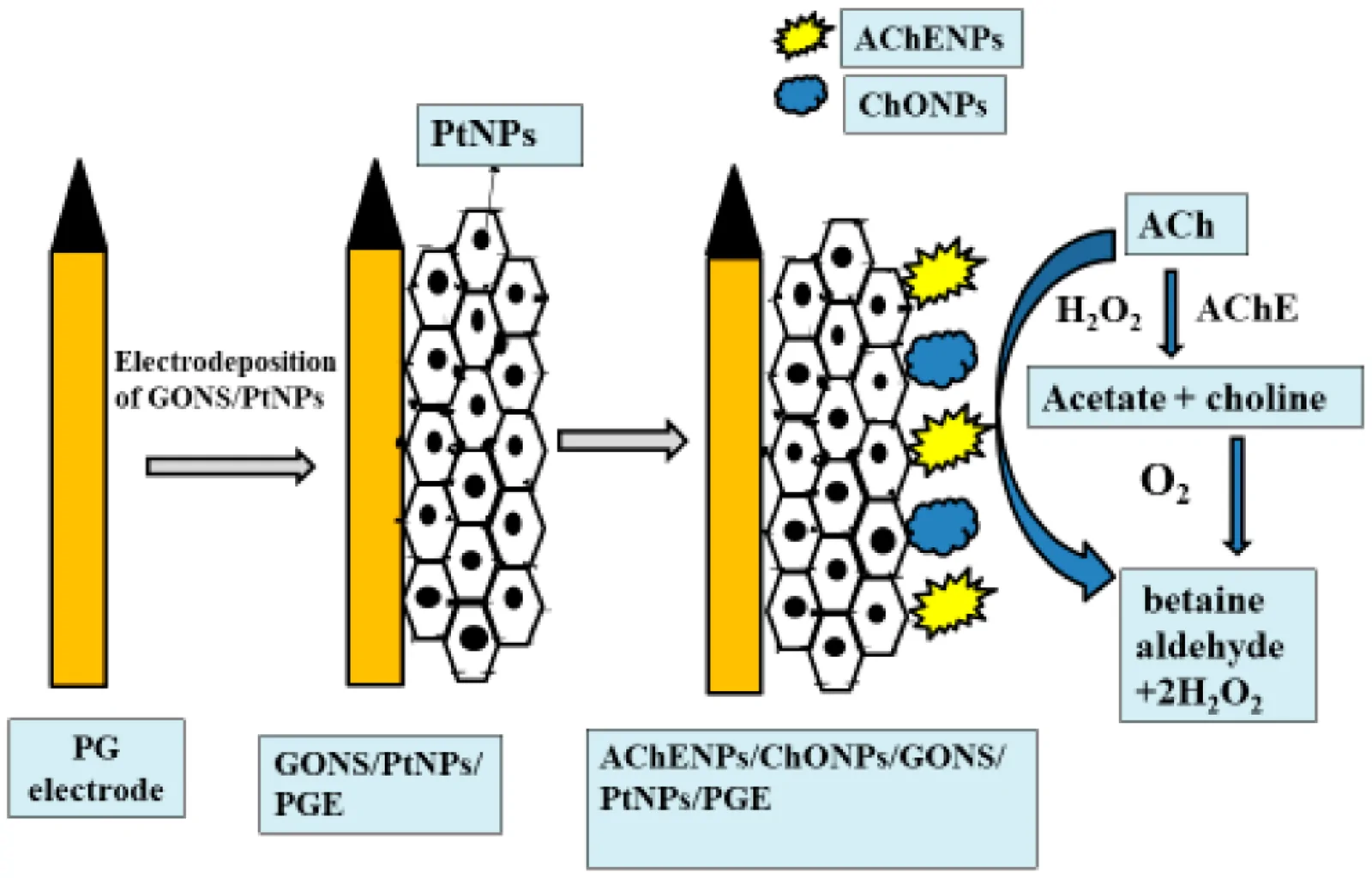
Schematic representation shows the construction of the ACh biosensor based on AChENPs/ChONPs/GONS/PtNPs/PGE. AChENPs, acetylcholinesterase nanoparticles; ChONPs, choline oxidase nanoparticles; GONS, graphene oxide nanosheets; PtNPs, platinum nanoparticles; PGE, pencil graphite electrode. Reproduced with permission from [22].

**Figure 3:**
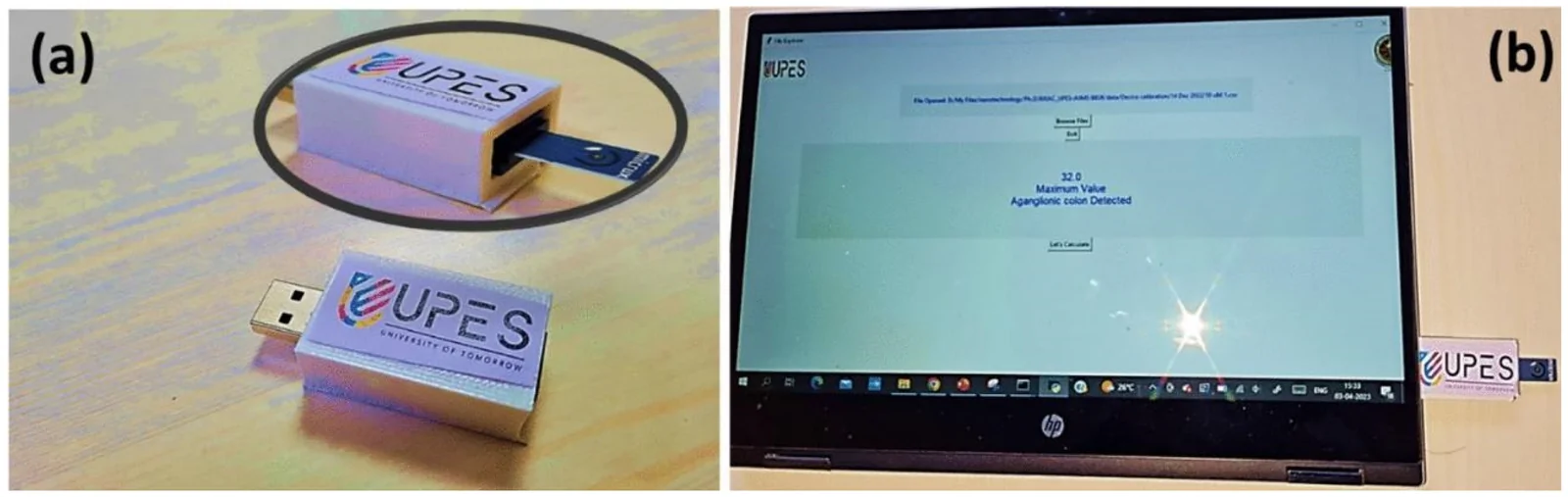
Example of a portable electrochemical biosensing system for intraoperative ACh measurement. (**a**) Portable potentiostat featuring a USB interface and sensor electrode insertion slot. (**b**) Integrated sensor electrode connected to a laptop for real-time electrochemical measurements and intraoperative bowel segment mapping. Reproduced with permission from [73].

**Figure 4:**
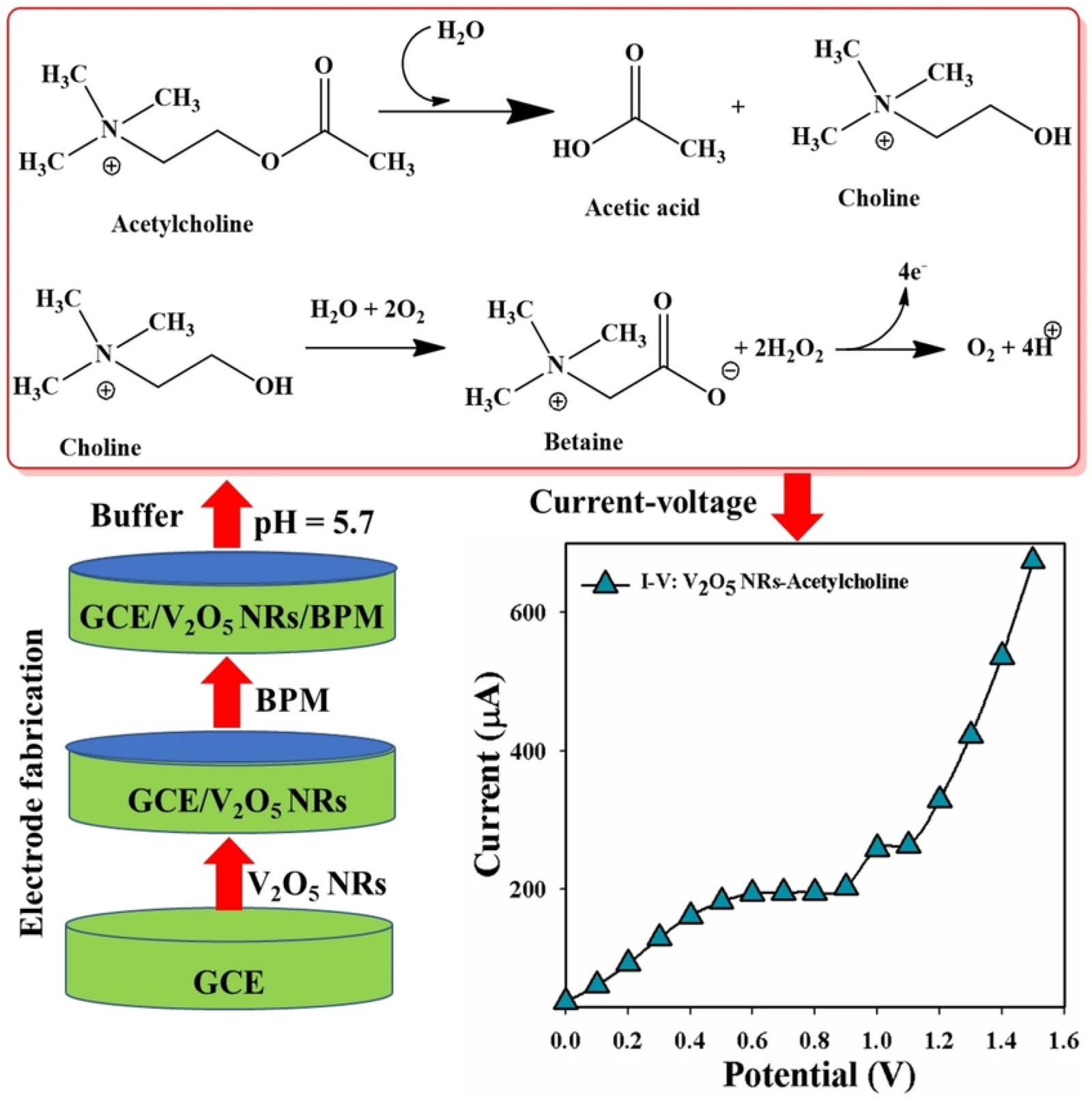
Schematic representation of the GCE/V_2_O_5_ NRs/BPM electrochemical sensing platform, illustrating electrode modification, ACh detection, and signal analysis based on current-voltage (I-V) measurements. Reproduced with permission from [69].

**Table 1:** Electrochemical sensors for ACh determination described in recent studies (2020–2026).

Study	Transductor	Detection Technique	Bioreceptor	Immobilization/Modification Strategy	Sample	Linear Range (LOD)	Assay/Response Time	Storage Stability (Days)
[66]	CPE	Amperometry	AChE, ChOx	PAMAM-Sal dendrimer (GA cross-linking)	Spiked synthetic blood	10^−2^–10 μM (5 nM)	-	3
[71]	GCE	Amperometry	-	CuMSs@carbon	Spiked human serum	10–100 μM (100 nM)	>90 min	NR
[69]	GCE	cyclic voltammetry	-	V_2_O_5_ nanorods-Nafion^®^	Spiked human, mouse, and rabbit serum	10^−4^–100 μM (0.011 nM)	8 s	NR
[22]	PGE	Amperometry	AChE, ChOx	PtNPs/ GONS nanocomposite. (adsorption)	Alzheimer patients’ serum	10^−3^–200 µM (1 nM)	3 s	180
[72]	AuE	EIS	AChE	DSP (covalent)	Rat brain tissue and rat blood	5.5–550 μM (NR)	3 h	NR
[67]	GCE	Amperometry	AChE, ChOx	Cu–N_4_/Cu–N_4_S-rGO (adsorption)	Spiked Human serum	0.02–0.1 μM (5 nM)	60 min	NR
[73]	SPE	Amperometry	AChE, ChOx	-	Spiked Human serum/Biopsies of Hirschsprung disease patients	NR (260 nM)	NR	NR
[68]	GCE	DPV	AChE, ChOx	PPy-CS nanofiber (covalent)	Spiked serum	10–1000 μM (5 µM)	<10 s	30 days
[70]	GFET	Electrolyte-gated GFET	AChE	GE-PBI (covalent)	Spiked buffer	0.1–1000 µM (NR)	NR	NR

Abbreviations: ACh: Acetylcholine; AChE: Acetylcholinesterase; AuE: gold electrode; ChOx: Choline oxidase; CPE: Carbon paste electrode; Cu–N_4_S-rGO: Copper nanosphere–reduced graphene oxide; CS: quitosan; CuMSs@carbon: copper microspheres; DPV: differential pulse voltammetry; DSP: Dithiobis (succinimidyl propionate); EIS: Electrochemical impedance spectroscopy; GA: glutaraldehyde; GE: graphene; GCE: Glassy carbon electrode; GFET: Graphene-field-effect transistor; GONS: porous graphene oxide nanosheet; LOD: Limit of detection; NR: not reported; PAMAM-Sal: Salicylaldehyde-functionalized poly (amidoamine) dendrimer; PBI: Polybenzimidazole; PGE: Pencil graphite electrode; PPy: Polypirrole; PtNPs: platinum nanoparticles; rGO: reduced graphene oxide; SPE: screen-printed electrode.

**Table 3:** Comparative overview of analytical performance, technological maturity, and future prospects of current ACh biosensing platforms.

Feature	Enzymatic Electrochemical Biosensors	Non-Enzymatic Electrochemical Biosensors	Optical Biosensors
Recognition principle	Enzyme-catalyzed hydrolysis of ACh (AChE/ChO)	Direct electrocatalytic oxidation using nanomaterials or nanozymes	Optical signal generation through fluorescence, SPR, colorimetry, or molecular probes
Biological recognition element	Required	Not required	Frequently required, depending on the sensing strategy
Analytical sensitivity	High-Excellent	High-Excellent	Variable, potentially excellent
Analytical selectivity	High	Moderate–High	High
Storage stability	Moderate, dependent on enzyme stability	Potentially high, but limited long-term evidence	Variable; often dependent on recognition element and optical probe stability
Resistance to biofouling	Moderate	Moderate	Platform-dependent
Fabrication complexity	Moderate–High	Moderate–High	High
Portability/Point-of-care compatibility	High	High	Moderate
Wearable/Implantable potential	Moderate–High	High	Moderate–High
Multiplexing capability	Moderate	Moderate	High
Validation in biological samples	Patient-derived samples reported	Mainly validated in spiked biological samples	Predominantly proof-of-concept studies
Translational maturity	Relatively advanced, but predominantly preclinical	Early preclinical development	Early preclinical development
Major strength	High sensitivity, specificity and well-established enzymatic recognition	Enzyme-free operation and potential for improved operational stability	Spatially resolved detection, multiplexing, and non-contact sensing
Current limitation	Dependence on enzyme immobilization and long-term stability	Potentially reduced selectivity in complex biological matrices	Limited biological/clinical validation and dependence on optical instrumentation
Representative future application	Point-of-care and biochemical monitoring	Continuous wearable and implantable neurochemical monitoring	Neuroimaging, multiplexed sensing, and mechanistic neuroscience studies

## Data Availability

The original contributions presented in this study are included in the article. Further inquiries can be directed at the corresponding author.

## Abbreviations

The following abbreviations are used in this manuscript:

ACh	Acetylcholine
AChE	Acetylcholinesterase
AChENPs	Acetylcholinesterase nanoparticles
AD	Alzheimer’s disease
AuE	Gold electrode
ChAT	Choline acetyltransferase
ChOx	Choline oxidase
ChONPs	Choline oxidase nanoparticles
CNS	Central nervous system
CPE	Carbon paste electrode
CS	Chitosan
CSF	Cerebrospinal fluid
Cu-NS-rGO	Copper nanosphere–reduced graphene oxide
CuMS	Copper microspheres
CV	Cyclic voltammetry
DPV	Differential pulse voltammetry
DSP	Dithiobis(succinimidyl propionate)
EIS	Electrochemical impedance spectroscopy
FAST	Flexible Acetylcholine Sensing Thread
GA	Glutaraldehyde
GCE	Glassy carbon electrode
GE	Graphene
GFET	Graphene field-effect transistor
GNRs	Gold nanorods
GONS	Graphene oxide nanosheets
GRAB-ACh	GPCR-activation-based acetylcholine sensor
HPLC	High-performance liquid chromatography
HPLC-ED	High-performance liquid chromatography coupled with electrochemical detection
LC	Liquid crystal
LC-MS	Liquid chromatography coupled with mass spectrometry
LOD	Limit of detection
MMT	Montmorillonite
MOF	Metal–organic framework
Myr	Myristoylcholine
N-CQDs	N-doped carbon quantum dots
NMR	Nuclear magnetic resonance
NR	not reported
NRs	Nanorods
PAMAM-Sal	Salicylaldehyde-functionalized poly(amidoamine) dendrimer
PBI	Polybenzimidazole
PET	Positron emission tomography
PGE	Pencil graphite electrode
PNS	Peripheral nervous system
POM	Polarized optical microscopy
PPy	Polypyrrole
PtNPs	Platinum nanoparticles
rGO	Reduced graphene oxide
SAM	Self-assembled monolayer
SERS	Surface-enhanced Raman spectroscopy
SPE	Screen-printed electrode
SPR	Surface plasmon resonance
